# Evaluation of Available Cognitive Tools Used to Measure Mild Cognitive Decline: A Scoping Review

**DOI:** 10.3390/nu13113974

**Published:** 2021-11-08

**Authors:** Chian Thong Chun, Kirsty Seward, Amanda Patterson, Alice Melton, Lesley MacDonald-Wicks

**Affiliations:** 1 School of Health Sciences, Faculty of Health and Medicine, University of Newcastle, University Drive, Callaghan, NSW 2308, Australia; nicole.thong@hotmail.com (C.T.C.); Kirsty.Seward@newcastle.edu.au (K.S.); Amanda.Patterson@newcastle.edu.au (A.P.); Alice.Melton@uon.edu.au (A.M.); 2 Priority Research Centre for Physical Activity and Nutrition, University of Newcastle, Callaghan, NSW 2308, Australia

**Keywords:** dementia, mild cognitive decline, cognitive decline, mild cognitive impairment, neuropsychological tests, neuropsychological battery, cognitive screening tool, cognition, older adults

## Abstract

Cognitive decline is a broad syndrome ranging from non-pathological/age-associated cognitive decline to pathological dementia. Mild cognitive impairment MCI) is defined as the stage of cognition that falls between normal ageing and dementia. Studies have found that early lifestyle interventions for MCI may delay its pathological progression. Hence, this review aims to determine the most efficient cognitive tools to discriminate mild cognitive decline in its early stages. After a systematic search of five online databases, a total of 52 different cognitive tools were identified. The performance of each tool was assessed by its psychometric properties, administration time and delivery method. The Montreal Cognitive Assessment (MoCA, *n* = 15), the Mini-Mental State Examination (MMSE, *n* = 14) and the Clock Drawing Test (CDT, *n* = 4) were most frequently cited in the literature. The preferable tools with all-round performance are the Six-item Cognitive Impairment Test (6CIT), MoCA (with the cut-offs of ≤24/22/19/15.5), MMSE (with the cut-off of ≤26) and the Hong Kong Brief Cognitive Test (HKBC). In addition, SAGE is recommended for a self-completed survey setting whilst a 4-point CDT is quick and easy to be added into other cognitive assessments. However, most tools were affected by age and education levels. Furthermore, optimal cut-off points need to be cautiously chosen while screening for MCI among different populations.

## 1. Introduction

Dementia is currently recognised as a global health priority, and is one of the major causes of disability amongst older adults [1,2]. Globally, there are 50 million people diagnosed with dementia, with a disease burden of AUD 1.4 trillion annually [1,2]. As the population continues to age, the worldwide prevalence of dementia is predicted to triple to 152 million people within the next three decades [3]. This will result in further costs for governments, communities, families and individuals. In addition, the medical, psychological and emotional impact on those with dementia and to caregivers/families is significant and detrimentally affects their quality of life [1]. 

Cognitive decline is a broad syndrome ranging from non-pathological/age-associated cognitive decline to pathological mild cognitive impairment, and further progression to dementia [4]. Mild cognitive impairment (MCI) is a term used to identify the stage of cognition that falls between normal ageing and dementia, defined as slight but measurable cognitive decline without the loss of functional ability [5,6,7]. Therefore, cognitive decline is recognised to occur through a mild and subtle manner onto a more comprehensive presentation; and its changes form a continuum [4]. Different from dementia, people with MCI can perform daily living activities independently with minimal aids or assistance [5]. Its onset is evident since middle age (age 45 to 49), but the failure to detect subtle cognitive changes has resulted in the delay of care among 27–81% of affected patients [8,9,10]. Detection can be unpredictable because each individual experiences different rates of decline [4]. In addition, research indicates that MCI is associated with heightened risk of progression to dementia as compared to individuals with more normal cognition [11]. 

Due to the poor prognosis implications, early detection of subtle cognitive changes is beneficial for practitioners to identify possible treatable causes or provide appropriate interventions. Currently, the clinical diagnosis of MCI is mainly determined by a physician’s best judgement [12,13]. Clinical characterisation methods including the Clinical Dementia Rating (CDR) scale, Petersen’s Criteria and the National Institute on Ageing-Alzheimer’s Association (NIA-AA) Criteria are frequently used in combination with laboratory and neurological tests to diagnose MCI [7]. These tests need to be administered by trained physicians and require extensive amounts of time. Hence, various brief cognitive tools have been introduced to detect cognitive decline as first-line screening methods [14]. A structured screening tool is required to be brief, easy to administer, have good psychometric properties, generalisable in elderly populations, and preferably able to be self-administered or conducted by non-health care professionals [14]. Many studies had evaluated and validated the dementia screening tests; however, there is limited research on MCI screening tools specifically. The most recent systematic review suggested that the Montreal Cognitive Assessment (MoCA) is the preferred tool for screening MCI in the primary care setting [14]. However, only a limited number of studies (14 articles) were included in this review [14]. There is also a lack of knowledge regarding the generalisability and usability of the tools in other settings and/or populations [14].

Disease-modifying therapy (DMT) for cognitive decline is currently a prioritised global research area to manage the rise in prevalence of cognitive decline and associated costs to society [15]. It is clear from clinical trials that there is a lack of pharmacological agents which are able to treat the underlying cause(s) or slow down the rate of cognitive decline [5]. Primarily, these pharmacological agents can only manage the symptoms by temporarily ameliorating memory and cognitive problems [5]. Hence, the emphasis of research has shifted to utilising lifestyle modifications as prevention or early treatment approaches. Several studies have shown a relationship between the development of cognitive decline and lifestyle-related risk factors [16]. Therefore, World Health Organisation guidelines recommend stakeholders to target modifiable lifestyle factors including improved nutrition and diet to diminish the risk [3,16]. This is supported by a recent systematic review which demonstrated that the modification of diet quality is a promising, yet long-term (more than 6 months) preventive measure to limit the progression of cognitive decline [17]. Even so, the lack of knowledge regarding the type and properties of cognitive tools remains one of the biggest barriers in research because the large range of tools used in studies makes comparison between studies difficult [17]. It is recommended that improved knowledge in the properties of cognitive assessment would help to elucidate the effectiveness of diet and nutrition in cognitive decline [17].

Therefore, the demand for easily administered, sensitive, specific and reliable cognitive tools to identify the early stages of subtle cognitive decline is high for several reasons. Firstly, identifying these tools can assist future researchers with selecting appropriate tools for the study design, and strengthen the ability to assess the effectiveness of interventions (both lifestyle and pharmacological) on the progression of cognitive impairment [18]. Secondly, health care practitioners can select these tools to assess an individual’s cognition and detect abnormal cognitive changes earlier, thus resulting in earlier intervention and improved patient outcomes [18]. 

In this study, we aimed to catalogue and assess the tools used to evaluate mild cognitive impairment and decline among healthy elderly populations. To achieve this, we considered multiple factors of the cognitive tools, including their psychometric performance and generalisability in different settings and/or populations. A scoping review instead of systematic review was chosen in order to include all the relevant information available and tools cited in the literature and to identify any gaps for future studies.

## 2. Materials and Methods 

### 2.1. Protocol and Registration

This protocol was developed using the methodological framework for scoping reviews proposed by Arksey and O’Malley (2005) [19] and further refined by using the Preferred Reporting Items for Systematic reviews and Meta-Analyses extension for Scoping Reviews (PRISMA-ScR) Checklist [20]. The protocol for this review was registered with the Open Science Framework: https://osf.io/tb3gc/ (accessed in 1 June 2020).

### 2.2. Eligibility Criteria

To be included in this review, papers need to be focused on the evaluation of screening and/or diagnostic performance of cognitive tools used to measure mild cognitive decline. Peer-reviewed journal papers were included if they were: in English language, assessed general healthy adult humans (>45 years, without any diagnosed health conditions or diseases) and evaluated the psychometric performance (i.e., specificity, sensitivity, validity, reliability) of cognitive tools. All quantitative study designs were eligible for inclusion. However, reviews and grey literature were excluded. Papers were excluded if they did not meet the above specified criteria or they focused on interventions rather than performance of cognitive tools. Tools that are not easily administered or are invasive (such as imaging tools or biomarkers) were also excluded. Moreover, papers published before 2015 were excluded to provide an up-to-date review on current literature. All papers had to be easily available to the research team at the time of the study, as time was limited due to the nature of the embedded honours program of the principal researcher. 

### 2.3. Information Sources and Search

Comprehensive literature searches for potentially relevant articles up until April 2020 were conducted in the following online databases: CINAHL (Ebsco), MEDLINE (Ovid), EMBASE (Ovid), PsycINFO (Ovid) and Cochrane. The search strategies were developed with the assistance of an experienced research librarian. The search strategy contained population, intervention and outcome terms. Searches were limited to adults aged 45 years and above as this is the age range in which mild cognitive decline presents [9]. The articles with publication dates before 2015 were excluded to provide an up-to-date review. The final search strategy for MEDLINE can be found in Supplementary Table S1. Similar search strategies were used while conducting searches in other identified databases. The final search results were exported into the EndNote X9 [21] referencing software. After removing the duplicates, the results were uploaded onto the online systematic review management system Covidence [22] for article screening purpose.

### 2.4. Selection of Sources of Evidence

**Figure 1:**
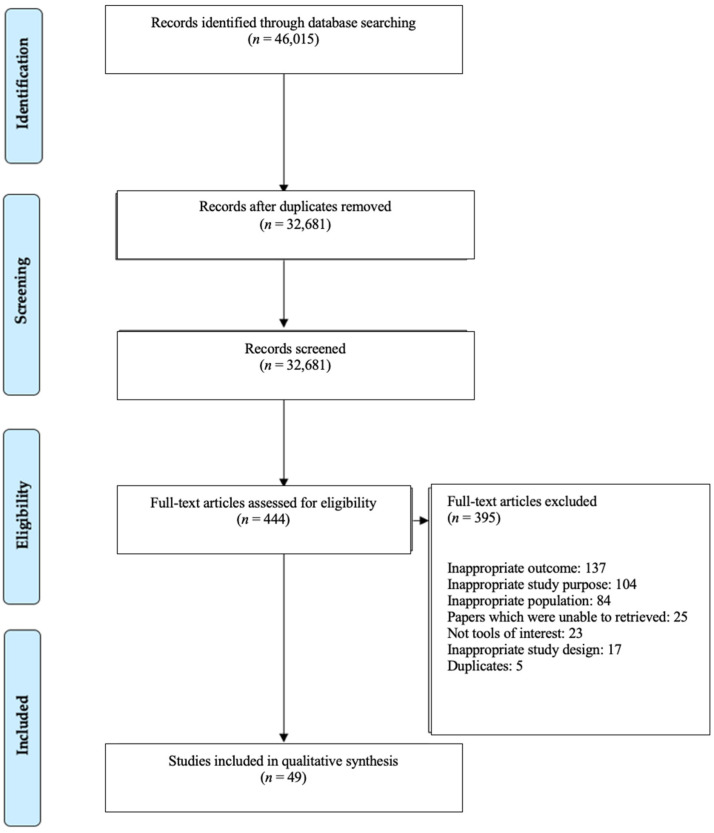
PRISMA flow chart for study selection process.

After removing duplicates from EndNote X9 [21] and Covidence [22], 32,681 publications were available for screening (Figure 1). Prior to screening, 3 reviewers (CTC, KS and AM) conducted screening trials and discussions on two occasions to increase consistency among reviewers. During the screening trials, CTC, KS and AM double screened 10 articles independently before discussions. After the mutual agreement of screening trial results, abstracts and titles of potentially relevant articles were single screened by CTC, KS or AM in Covidence [22]. Full-text screening and discussions as above were conducted again prior to data extraction. Relevant full-text articles (*n* = 444) were single screened by CTC, KS or AM against the inclusion criteria, with the reason for exclusion recorded. All included full-text papers (*n* = 49) underwent data extraction.

### 2.5. Data Charting Process and Data Items

CTC designed a standardised data-charting form (a customised spreadsheet) under supervision to chart data from eligible studies and to determine the appropriate variables to extract. The included variables in the spreadsheet were study characteristics (author, year, country of origin), characteristics of tools (name of the tool, the version of tool, range of the scores/points, cut-off point to detect mild cognitive decline, administration method and the duration of administration), study design, study population (age, %female, education level), settings, the psychometric performance of tools (including sensitivity, specificity, reliability and validity in detecting mild cognitive decline), factors that may affect the performance of the cognitive tool and the comparison standard(s) in the validation studies. 

CTC charted the data in the data charting form under supervision. LMW checked the extracted data. AM hand-search the information if there was missing data in the spreadsheet. KS double-checked 10% of the extracted data. Reviewers iteratively updated the data-charting form before synthesising the results. 

### 2.6. Synthesis of Results

By using the standardised data-charting form, all results were summarised and synthesised after discussions with all reviewers. By using the Preferred Reporting Items for Systematic reviews and Meta-Analyses (PRISMA) flowchart, reviewers documented the screening methods and recorded the quantity of included and excluded studies in this review (Figure 1). Additionally, by using the coding system, reviewers counted the frequency that each tool cited in included papers to catalogue which tool had the most frequent research done on its performance. 

Regarding the psychometric properties, validity was charted as the Sensitivity (Sn), Specificity (Sp), Area Under the Curve (AUC), Positive Predictive Value (PPV) and Negative Predictive Value (NPV). Sn is the ability of a tool to correctly classify an individual as having ‘mild cognitive decline’, whereas Sp is the ability of a tool to correctly classify an individual as ‘without mild cognitive decline’ [23]. AUC is an overall measurement of validity performance of a screening/diagnostic test [13]. PPV is the percentage of patients with a positive test who actually have ‘mild cognitive decline’; whereas NPV is the percentage of patients with a negative test who actually do not have ‘mild cognitive decline’ [23]. All the above properties were charted as percentages, with the closeness to 100% being higher respective validity. Reliability of a tool was identified based on its performance on all reliability tests used in the included studies. Interpretation of the above properties is presented in Table 1. By referencing with other validity studies, reviewers interpreted the psychometric properties based on the criteria developed by researchers’ consensus [13,24]. To be classed as good, the cognitive tool has to achieve the below criteria: good to excellent validity, good reliability, short administration time of ≤15 min whilst being able to be self-administered or conducted by non-health care professionals [14]. Hence, reviewers assessed the performance of cognitive tools using the above appraisal format. 

Lastly, a narrative synthesis of results was developed to assess and evaluate the characteristics and psychometric properties of each of the identified cognitive tools based on the data charting form and the criteria (Table 1).

## 3. Results

### 3.1. Study Selection

In total, 46,015 articles published in the five-year period (2015 to April 2020) were retrieved. After removing duplicate articles, 32,681 articles were screened in Covidence [22], with another 395 articles excluded due to inappropriate outcomes (*n* = 137), inappropriate study purpose (*n* = 104), inappropriate population (*n* = 84), papers which were unable to be retrieved (*n* = 25), not tools of interest (*n* = 23), inappropriate study design (*n* = 17) and duplicated articles (*n* = 5). After evaluating the full text, 49 articles met inclusion criteria and were included in this review.

### 3.2. Study Characteristics

Key characteristics of the 49 included articles can be found in Table 2. Considerable variations were found between studies for country, participant’s characteristics, studied cognitive tools and their comparison standard(s). The majority of studies were conducted in Asian countries (*n* = 17) [25,26,27,28,29,30,31,32,33,34,35,36,37,38,39,40,41], followed by European countries (*n* = 13) [42,43,44,45,46,47,48,49,50,51,52,53,54] and the Unites States (*n* = 7) [55,56,57,58,59,60,61]. The remaining studies came from Brazil (*n* = 3) [62,63,64], Australia (*n* = 2) [65,66], Greece (*n* = 2) [67,68], Argentina (*n* = 1) [69], unclear origin (*n* = 2) [70,71], Cuba (*n* = 1) [72] and Turkey (*n* = 1) [73]. In terms of study design, most included articles were cross-sectional (*n* = 33) [25,26,27,28,29,31,32,34,35,37,38,39,40,41,42,43,44,45,46,47,49,53,54,63,65,66,67,68,69,70,71,72,73] and cohort studies (*n* = 14) [30,33,36,48,50,51,52,55,56,57,58,61,62,64]. The characteristics for participants in each study were similar, with the age ranging from 50 to 95 years and the proportion of females ranging from 33 to 87%. Participants with low, average and high levels of education were included. To evaluate the psychometric performance of tools, studies used various validated comparison standards including the Clinical Dementia Rating (CDR) [26,32,56,65], the Mini-Mental State Examination (MMSE) [25,42,45,46,49], Petersen’s criteria [29,36,53,57,64,71,73], National Institute on Ageing-Alzheimer’s Association (NIA-AA) criteria [40,44,47,50,70], brief cognitive tests [59,67], clinical consensus by health professionals [61], Magnetic Resonance Imaging (MRI) scans, Diagnostic and Statistical Manual of Mental Disorders criteria (DSM) [27], other methods [51,60,63,68,72], or a combination of the above standards [28,30,31,33,34,35,37,38,39,41,43,48,52,55,56,62,66,69,72] to classify participants as ‘mild cognitive decline’ or ‘without mild cognitive decline’.

### 3.3. Cognitive Tools for Mild Cognitive Decline

A total of 52 different cognitive tools used to detect cognitive decline were catalogued and assessed in this review (Table 3). The Montreal Cognitive Assessment (MoCA) (*n* = 15) [26,27,28,29,31,32,34,36,44,49,56,61,63,65,73] and MMSE (*n* = 14) [26,27,28,29,32,34,36,40,50,53,57,66,72,73] followed by the Clock Drawing Test (CDT) (*n* = 4) [47,50,51,54] were most frequently cited in the literature. The other 49 tools were only studied in a limited number of articles (1 to 2 studies each). All of the tools were studied in clinical context and were applied in primary care and/or community settings. Most of the tools need to be administered by health care professionals (*n* = 14) [28,32,35,36,38,46,47,49,54,58,59,62,63,64,65,67,72,73] or trained personnel (*n* = 12) [26,31,33,39,40,41,44,53,65,68,70,72]. The remaining tools can be conducted by untrained examiners (*n* = 6) [27,29,42,45,51] or self-administered (*n* = 6) [30,43,53,58,60,62]. Among the self-administered tools, the Hong Kong–Vigilance and Memory Test (HK-VMT) [30] and the Self-Administered Gerocognitive Examination (SAGE) [60] can be administered via electronic devices.

### 3.4. Psychometric Performance of Included Cognitive Tools

Table 4 collates the available version(s), cut-off point(s), and psychometric performance (validity and reliability), factors which affect the performance and the administration time of the cognitive tools. Table 5 summarises all the data for the performance of the cognitive tools compared with the pre-identified criteria on the tools overall performance. Based on the researchers’ appraisal, there are several cognitive tools that achieved the status of good cognitive tool, including the Six-item Cognitive Impairment Test (6CIT), MoCA (with the cut-offs of ≤24/22/19/15.5), MMSE (with the cut-off of ≤26) and the Hong Kong Brief Cognitive Test (HKBC). 

These tools provided good to excellent validity and reliability in detecting people with mild cognitive decline within 15 min of administration time. In addition, they do not require health care professionals to administer. However, education levels, age, gender and emotional status can affect the performance of these cognitive tools. For instance, the performance of 11 tools were found to be associated with education [27,28,29,30,31,36,42,50,53,67,68,72] while the results of 10 tools were associated with age [28,36,39,41,45,50,67,68,72]. In addition, a briefer, revised or translated version which can better accommodate the settings of specific populations was also available for most of the tools [25,26,27,29,31,32,38,40,41,42,44,45,46,48,49,52,53,55,58,59,60,62,63,64,66,68,69,72,73].

## 4. Discussion

This scoping review collates a comprehensive list of brief cognitive tools used to measure mild cognitive decline in healthy elderly populations. To achieve effective screening outcomes, the brief cognitive tools are required to have good to excellent psychometric properties, short administration time and can be self-administered or administered by non-health care professionals [14,24]. 

Similar to recent systematic reviews, MoCA, MMSE and CDT are the most commonly used cognitive assessment tools in screening mild cognitive decline [14,74]. Based on our critical evaluation (Table 5), the ideal screening tools with versatile performance are 6CIT [42], MoCA (with the cut-offs of ≤24/22/19/15.5) [26,27,28,31,32,44,49,56], MMSE (with the cut-off of ≤26) [26,27,28,50,72] and HKBC [27]. The remaining 48 tools have suboptimal performance or insufficient information in any of these criteria: psychometric properties, administration time or administration methods. All of these tools are suitable to use in community or primary care settings.

Among these ideal screening tools, HKBC has the highest validity and reliability in identifying the earliest stages of subtle cognitive decline [27]. However, it was only validated in Hong Kong with a limited number of studies, and might not be generalisable among other populations.

MMSE is the most recognised brief cognitive tool which is frequently used in measuring cognitive impairment in clinical, research and community settings [75]. However, as supported by multiple systematic reviews and meta-analysis, MoCA can detect the subtle changes in cognitive capacity better than MMSE [14,75,76]. Studies proposed that there are several features in MoCA’s design that can potentially explain its superior sensitivity in MCI detection [77]. As compared to MMSE, MoCA’s assessment tasks includes more words, fewer learning trials, and a longer delay before the memory recall test [77]. MCI participants can be mildly impaired in their executive functions, complex visuospatial processing and the higher-level language abilities [77]. Thus, MoCA with more diverse and demanding tasks can better distinguish the changes in the above components than MMSE [77].

Even so, both MoCA and MMSE are recommended as the widely generalisable cognitive tools with all-round performance. They have been adapted and validated in different versions to minimise the effect of language and culture on their psychometric performance. Both tools can be administered by trained or untrained personnel in multiple health care settings such as hospital, primary care and the community. However, not all cut-off points provide high psychometric performances in screening mild cognitive decline. Different cut-off scores have also been published when the tests are modified to suit the local culture [74]. Hence, optimal cut-off points need to be carefully chosen while interpreting these results. Nonetheless, the presence of educational bias remains a concern while administering MoCA and MMSE and this was supported by a systematic review by Roshaslina Rosli et al. [74]. The impact of education may result in inappropriate referral due to the overestimation of the prevalence of mild cognitive decline [74]. To address this issue, MoCA-B is an modified version of MoCA which was designed to be less dependant on literacy levels [32]. Additional studies in this area may be beneficial for future use and development of the tools. Alternatively, Visual Cognitive Assessment Test (VCAT) is not affected by languages or cultural background, overcoming the common barriers for most cognitive tools including MoCA and MMSE [33,35]. It is designed to be a visual-based cognitive tool to reduce the language demands [35]. Only the instructions, but not the test components require translation [35]. Based on our appraisal, the only criteria resulting in its exclusion from the ‘good cognitive tool’ category was the slightly lengthy administration time (15 to 20 min) for a brief cognitive tool [33].

To detect mild cognitive decline in surveys, self-completed tools such as the Dementia Screening Interview (AD8), SAGE, the Everyday Memory Questionnaire (EMQ), the Cognitive Change Questionnaire (CCQ), HK-VMT and Test Your Memory (TYM) can be suitable. Among these self-administered tools, SAGE has the best validity and reliability and is also validated to be conduct via electronic devices [60]. From our review, there are some very brief cognitive tools which required less than 5 min to deliver. 6CIT is the preferable very brief cognitive tool with versatile properties [42]. However, it was only validated against MMSE which is not a true gold standard in diagnosing MCI [42]. A 4-point CDT only requires less than 2 min to conduct [50]. Its only limitation is the fair to good validity while screening MCI. Thus, CDT may be beneficial to use in combination with other screening tools without adding a significant amount of administration time. In addition, a short-form Brief Cognitive Assessment Tool (BCAT) is also valid and reliable to be conducted by professional personnel within 3 to 4 min [58].

Interestingly, the level of psychometric performance can be different while screening different types of MCI. There are generally two subtypes of MCI, which are amnestic MCI (a-MCI) and non-amnestic MCI (na-MCI) [78,79]. Research has shown that there are structural differences in brain tissues among different MCI subtypes and these pathological changes affect different cognitive components [80]. Thus, people with a-MCI have impaired memory whereas na-MCI affects people’s thinking skills other than memory [78,79]. Hence, cognitive tests which assessed different domains may have different performance in identifying each MCI subtype. For instance, Short Test of Mental Status (STMS) has higher validity in discriminating na-MCI as compared to a-MCI which is potentially due to its assessment properties of having a larger domain in assessing memory rather than other cognitive skills [61,81]. Therefore, future studies are recommended to further validate the MCI screening tools’ performance in discriminating different subtypes of MCI. Additional studies were also required to further validate the cut-off points and psychometric performance of the included brief cognitive tools in this review.

The limited available studies and data among included articles remains the biggest limitation to our review. The exclusion of studies before 2015, grey literature and non-English studies may limit some of the information relevant to this review. To make this review more feasible within the honours program limitation, the optional critical appraisal of study quality was not conducted in this review. Despite these limitations, this is a thorough scoping review and has collated a large number of studies from the previous 5 years. Studies from various countries were included, which allowed us to catalogue the brief cognitive tools used in worldwide populations and across a variety of settings. Substantial work was undertaken to evaluate each of the tools used in measuring mild cognitive decline.

## 5. Conclusions

Based on our review, there were 52 different tools available to discriminate mild cognitive decline among healthy elderly populations. 6CIT [42], MoCA (with the cut-offs of ≤24/22/19/15.5) [28,32,34,35,44,46,49,60], MMSE (with the cut-off of ≤26) [26,27,28,50,72] and HKBC [27] are good at discriminating the subtle cognitive changes as a result of MCI. They have versatile performance in terms of their psychometric properties, administration time and delivery methods. In addition, MoCA and MMSE have been modified into various versions to be generalisable in multiple populations. To detect subtle cognitive changes in surveys, SAGE is recommended, and it can also be administered digitally. A 4-point CDT is quick and easy to be added into other cognitive screening tests while assessing MCI. However, suitable cut-off points need to be further studied to validate performance as a mild cognitive decline screening test.

The lack of thorough evaluation of cognitive tools in identifying MCI appears to be a challenge among clinical and research settings. The aim of this review was to catalogue and assess the tools used to evaluate mild cognitive decline among healthy elderly populations, and to identify gaps in the literature which might guide future research in this area. This review advocates additional research being needed to recommend the best MCI cognitive screening tools among different populations and environments.

## Figures and Tables

**Table 1:** Validity criteria for cognitive tools.

Criteria *	Interpretation	Range (%)
Sn and Sp	Excellent	91–100
	Good	76–90
	Fair	50–75
	Poor	<50
AUC	Excellent	91–100
	Good	81–90
	Fair	71–80
	Poor	<70
PPV and NPV	Excellent	91–100
	Good	76–90
	Fair	50–75
	Poor	<50

* The criteria for Sn, Sp, PPV and NPV were decided based on researchers’ consensus. The criterion for AUC was adapted from Safari S et al. [13].

**Table 2:** Included studies.

No.	Authors, Year, Country	Study Design	Participants Characteristics			Cognitive Tool	Comparison Standard
			Age (Mean ± SD or Range)	% Female	Education Years (Mean ± SD or Range)		
1	Apostolo JLA et al., 2018, Portugal [42]	Cross-sectional	67.7 ± 9.7	70.4	30.7% 0–2 years, 43.3% 3–6 years, 26% 7–18 years	6CIT	MMSE
2	Avila-Villanueva M et al., 2016, Spain [43]	Cross-sectional	74.07 ± 3.8	63	11.15 ± 6.69	EMQ	CDR, NIA-AA criteria
3	Baerresen KM et al., 2015, US [55]	Cohort	60.84 ± 10.76	60	16.67 ± 2.94	BSRT, RCFT, TMT	Rigorous diagnostic methods: MRI scan, clinical consensus of neurology, geriatric psychiatry, neuropsychology and radiology staff
4	Bartos A et at., 2018, Czech Republic [44]	Cross-sectional	70 ± 8	59	12–17	MoCA	NIA-AA criteria
5	Bouman Z et al., 2015 Netherlands [45]	Cross-sectional	76.6 ± 5.9	~46	~66% low level, 19% average level, 16% high level	BCSE	MMSE
6	Broche-Perez Y et al., 2018, Cuba [72]	Cross-sectional	73.28 ± 7.16	~67	9.82 ± 4.23	ACE, MMSE	Petersen’s criteria, CDR
7	Charernboon T, 2019, Thailand [25]	Cross-sectional	64.9 ± 6.5	76.7	10.2 ± 4.9	ACE	Thai version of MMSE
8	Chen K-L et al., 2016, China [26]	Cross-sectional	68.2 ± 9.1	~66	4.8 ± 1.7	MMSE, MoCA	CDR
9	Chipi E et al., 2017, Italy [46]	Cross-sectional	70.9 ± 5.1	61.2	11.5 ± 4.5	CFI	MMSE
10	Chiu HF et al., 2017, Hong Kong [27]	Cross-sectional	75.4 ± 6.6	56.6	6.5 ± 3.8	HKBC, MoCA, MMSE	DSM-5
11	Chiu P et al., 2019, Taiwan [28]	Cross-sectional	67.8 ± 10.7	47.2	6.9 ± 5.1	MMSE, NMD-12, MoCA, IADL, AD8, CASI, NPI	NIA-AA criteria, CDR
12	Chu L et al., 2015, Hong Kong [29]	Cross-sectional	72.2 ± 6.1	87	6.97 ± 4.69	MMSE	Petersen’s criteria
13	Clarnette R et al., 2016, Australia [65]	Cross-sectional	50–95	52	4–21	Qmci, MoCA	CDR
14	Damin A et al., 2015 Brazil [62]	Cohort	68.27 ± 7.34	N/A	7.48 ± 4.48	CCQ	MMSE, CAMCog, CDR and the brief cognitive screening battery
15	Duro D et al., 2018, Portugal [47]	Cross-sectional	69.47 ± 8.89	63.5	6.69 ± 4.14	CDT	NIA-AA criteria
16	Freedman M et al., 2018 [70]	Cross-sectional	75.3 ± 7.9	~67	15.02 ± 3.2	TorCA	NIA-AA criteria
17	Fung AW-T et al., 2018, Hong Kong [30]	Cohort	68.8 ± 6.3	58.4	9.8 ± 4.8	HK-VMT	Combined clinical and cognitive criteria suitable for local older population, CDR
18	Georgakis MK et al., 2017, Greece [67]	Cross-sectional	74.3 ± 6.6	51.6	4.5 ± 2.6	TICS	5-objects test
19	Heyanka D et al., 2015 [71]	Cross-sectional	71.5 ± 7.5	~43	14.8 ± 3.2	RBANS	Petersen’s criteria
20	Huang L et al., 2018, China [31]	Cross-sectional	65.71 ± 8.10	~56	12.78 ± 2.74	RCFT, MoCA, VOSP, BNT, STT, JLO, ST	Petersen’s criteria, CDR
21	Iatraki E et al., 2017, Greece [68]	Cross-sectional	71.0 ± 6.9	64.6	6.4 ± 3.1	TYM, GPCog	Unclear
22	Julayanont P et al., 2015, Thailand [32]	Cross-sectional	66.6 ± 6.7	84	3.6 ± 1.1	MoCA, MMSE	CDR global
23	Khandiah N et al., 2015, Singapore [33]	Cohort	67.8 ± 8.86	46.1	10.5 ± 6.0	VCAT	Petersen’s criteria, CDR, NIA-AA criteria
24	Phua A et al., 2017, Singapore [34]	Cross-sectional	66.8 ± 5.5	62	9.3 ± 4.9	MoCA, MMSE	DSM-IV, CDR global
25	Krishnan K et al., 2016, US [56]	Cohort	58–77	64	15.2 ± 2.7	MoCA	History, clinical examination, CDR, and a comprehensive neuropsychological battery based on published criteria
26	Lee S et al., 2016, Australia [66]	Cross-sectional	Median 73	53	Median 14	CVLT, The Envolope Task, PRMQ, Single-item Memory Scale, MMSE	HVLT-R, Logical Memory, Wechsler Memory Scale Third Edition, Verbal Paired Associates, Wechsler Memory Scale Fourth Edition, RCFT, CDR, ADFACS, NINCDS-ADRDA criteria, MMSE
27	Lemos R et al., 2016, Portugal [48]	Cohort	70.22 ± 7.65	52.5	7.7 ± 5.01	FCSRT	MMSE, CDR
28	Low A et al., 2019, Singapore [35]	Cross-sectional	61.47 ± 7.19	70	12.36 ± 3.76	VCAT	NIA-AA criteria, CDR, MRI scan
29	Malek-Ahmadi M et al., 2015, US [57]	Longitudinal Cohort	81.70 ± 7.25	~48	14.74 ± 2.54	MMSE, AQ, FAQ	Petersen’s criteria
30	Mansbach W et al., 2016, US [58]	Cohort	82.33 ± 9.15	64	84% at least 12 years education	BCAT, AD8	Unclear, diagnosed by licensed psychologist’s evaluations
31	Mellor D et al., 2016, China [36]	Cohort	72.54 ± 8.40	57.9	9.12 ± 4.36	MoCA, MMSE	Petersen’s criteria
32	Mitchell J et al., 2015, US [59]	Case–control	75.9 ± 8.5	50.9	15.2 ± 2.9	FAQ, DSRS, CWLT, BADLS	WMS-III Logical Memory test or the CERAD Word List
33	Ni J et al., 2015, China [37]	Cross-sectional	62.57 ± 8.61	~59	12.04 ± 3.34	DSR	History and physical exams, MMSE, story recall (immediate and 30 min delayed), CDR, ADL
34	Park J et al., 2018, South Korea [38]	Cross-sectional	74.93 ± 6.96	56.3	5.83 ± 4.52	mSTS-MCI	MoCA-K, MMSE-K, neuropsychological battery (Rey Auditory Verbal Learning Test and Delayed Visual Reproduction and Logical Memory, two subtests of the Wechsler Memory Scale)
35	Pinto T et al., 2019, Brazil [63]	Cross-sectional	73.9 ± 6.2	76.4	10.9 ± 4.4	MoCA	Statistically compared
36	Pirrotta F et al., 2014, Italy [49]	Cross-sectional	70.5 ± 11.5	58.2	8.1 ± 4.6	MoCA	MMSE
37	Radanovic M et al., 2017, Brazil [64]	Cohort	~68.7 ± 5.85	~79	~10.35 ± 2.45	CAMCog	Petersen’s criteria
38	Rakusa M et al., 2018, Slovenia [50]	Cohort	Median 74	N/A	65% Secondary school, 23% University, 12% Primary School	MMSE, CDT	NIA-AA criteria
39	Ricci M et al., 2016, Italy [51]	Cohort	73.3 ± 6.9	N/A	7.2 ± 4.2	CDT	NINCDS- ADRDA criteria
40	Roman F et al., 2016, Argentina [69]	Cross-sectional	67.5 ± 8.3	N/A	11.5 ± 4.1	MBT	Spanish Version of MMSE, CDT, Signoret Verbal Memory Battery, TMT, VF, Spanish Version of BNT, and the Digit Span forward and backward
41	Scharre D et al., 2017, US [60]	Investigational	75.2 ± 7.3	67	15.1 ± 2.7	SAGE	Unclear
42	Serna A et al., 2015, Spain [52]	Cohort	78.10 ± 5.04	59.3	64.2% illiteracy/read and write, 35.8% primary/secondary or higher	Semantic Fluency/VF, Logical Memory	International Work Group criteria, MMSE
43	Townley R et al., 2019 US [61]	Cohort	~72.4 ± 8.95	47–51	~ 15.05 ± 2.65	STMS, MoCA	Clinical consensus
44	Van de Zande E et al., 2017, Netherlands [53]	Cross-sectional	73.05 ± 8.62	~52	10.34 ± 3.66	MMSE, TYM	Petersen’s criteria
45	Vyhnálek M et al., 2016, Czech Republic [54]	Cross-sectional	71.20 ± 6.77	~64	15.30 ± 2.95	CDT	CDR
46	Feng X et al., 2017, China [39]	Cross-sectional	65.99 ± 10.45	62.59	2.88% 0 years, 7.19% 1–6 years, 51.08% 7–12 years, 38.85% ≥12 years	DMS48	Chinese Version of MMSE, MoCA, CDR, NIA-AA criteria
47	Xu F et al., 2019, China [40]	Cross-sectional	82.87 ± 3.134	33.4	62.8% having bachelor’s degrees	MMSE, GPCog	NIA-AA criteria
48	Yavuz B et al., 2017 Turkey [73]	Cross-sectional	75.4 ± 6.9	65	0–21 (Median 5)	MMSE, Qmci	Petersen’s criteria
49	Zainal N et al., 2016, Singapore [41]	Cross-sectional	61.81 ± 6.96	68.8	11.70 ± 3.13	ADAS-Cog	Petersen’s criteria, CDR

6 CIT: Six-item Cognitive Impairment Test; MMSE: Mini-Mental State Examination; EMQ: Everyday Memory Questionnaire; CDR: Clinical Dementia Rating; NIA-AA: National Institute on Aging-Alzheimer’s Association; BSRT: Buschke Simple Reaction Time; RCFT: Rey–Osterrieth Complex Figure Test; TMT: Trail Making Test; MRI: Magnetic Resonance Imaging; MoCA: Montreal Cognitive Assessment; BCSE: Brief Cognitive Status Exam; ACE: Addenbrooke’s Cognitive Examination. Abbreviations list for Table 2: CFI: Cognitive Function Instrument; HKBC: Hong Kong Brief Cognitive Test; DSM-5: Diagnostic and Statistical Manual of Mental Disorders, 5th Edition; NMD-12: Normal-MCI-Dementia 12 Questionnaire; IADL: Instrumental Activities of Daily Living; AD8: Dementia Screening Interview; CASI: Cognitive Abilities Screening Instrument; NPI: Neuropsychological Inventory; Qmci: Quick Mild Cognitive Impairment; CCQ: Cognitive Change Questionnaire; CAMCog: Cambridge Cognitive Examination; TorCA: Toronto Cognitive Assessment; HK-VMT: Hong Kong—Vigilance and Memory Test; TICS: Telephone Interview for Cognitive Status; RBANS: Repeatable Battery for the Assessment of Neuropsychological Status; VOSP: Visual Object and Space Perception; BNT: Boston Naming Test; STT: Shape Trail Test; JLO: Judgment of Line Orientation; ST: Similarity Test; TYM: Test Your Memory; GPCog: General Practitioner assessment of Cognition; DSM-IV: Diagnostic and Statistical Manual of Mental Disorders, 4th Edition; CVLT: California Verbal Learning Test; PRMQ: Prospective and Retrospective Memory Questionnaire; HVLT-R: Hopkins Verbal Learning Test—Revised; ADFACS: Alzheimer’s Disease Functional Assessment and Change Scale; NINCDS-ADRDA: National Institute of Neurological and Communicative Disorders and Stroke and the Alzheimer’s Disease and Related Disorders Association; FCSRT: Free and Cued Selective Reminding Test; VCAT: Visual Cognitive Assessment Test; AQ: Alzheimer’s Questionnaire; FAQ: Functional Activities Questionnaire; BCAT: Brief Cognitive Assessment Tool; BLAT: Blind Learning Aptitude Test; DSRS: Severity Rating Scale; CWLT: CERAD Word List Memory Test; BADLS: Bristol Activities of Daily Living Scale; DSR: Delayed Story Recall; WMS-III: Wechsler Memory Scale-3rd Edition; CERAD: Consortium to Establish a Registry for Alzheimer’s Disease; ADL: Activities of Daily Living; mSTS-MCI: Mobile Screening Test System for screening Mild Cognitive Impairment; MoCA-K: Korean version of MoCA; MMSE-K: Korean version of MMSE; CDT: Clock Drawing Test; MBT: Memory Binding Test; VF: Verbal Fluency; SAGE: Self-Administered Gerocognitive Examination; STMS: Short Test of Mental Status; DMS48: Delayed Matching-to-Sample Task 48; ADAS-Cog: Alzheimer’s Disease Assessment Scale-Cognitive subscale.

**Table 3:** Included Tools and Its Study Characteristics.

No.	Cognitive Tool	Article No.	Authors, Year, Country	Settings	Administration Method
1	6CIT	1	Apostolo JLA et al., 2018, Portugal [42]	Community, Primary health care units	By untrained examiner (post-graduate student)
2	EMQ	1	Avila-Villanueva M et al., 2016, Spain [43]	Community	Self-administered
3	BSRT	2	Baerresen KM et al., 2015, US [55]	Community	NR
4	RCFT	2	Baerresen KM et al., 2015, US [55]	Community	NR
		2	Huang L et al., 2018, China [31]	Memory Clinic	By trained examiner
5	TMT	4	Baerresen KM et al., 2015, US [55]	Community	NR
6	MoCA	8	Bartos A et at., 2018, Czech Republic [44]	Community	By trained examiner
		10	Chen K-L et al., 2016, China [26]	Hospital	By trained examiner
		12	Chiu HF et al., 2017, Hong Kong [27]	Community	By untrained examiner (research assistant)
		13	Chu L et al., 2015, Hong Kong [29]	Memory Clinic, Community	By examiner
6	MoCA	13	Clarnette R et al., 2016, Australia [65]	Geriatrics Clinic	By trained professionals (geriatrician)
		22	Julayanont P et al., 2015, Thailand [32]	Community Hospital	By trained professionals (nurse with expertise in cognitive assessment)
		24	Phua A et al., 2017, Singapore [34]	Memory Clinic	NR
		25	Krishnan K et al., 2016, US [56]	Community, Clinical Care	NR
		31	Mellor D et al., 2016, China [36]	Community	By trained professionals (psychologist or attending level psychiatrist)
		35	Pinto T et al., 2019, Brazil [63]	Health Care Centres	By trained professionals (neurologist researcher)
		36	Pirrotta F et al., 2014, Italy [49]	Clinical, Research	By trained professionals (psychologist)
		43	Townley R et al., 2019 US [61]	Community	NR
		48	Yavuz B et al., 2017, Turkey [73]	Geriatrics Clinic	By trained professionals (psychologist)
		11	Chiu P et al., 2019, Taiwan [28]	Health Care Centres	By professionals (neuropsychologist)
		20	Huang L et al., 2018, China [31]	Memory Clinic	By trained examiner
7	BCSE	5	Bouman Z et al., 2015 Netherlands [45]	Memory Clinic	By untrained examiner
8	ACE	6	Broche-Perez Y et al., 2018, Cuba [72]	Primary Care Community Centre: nursing homes (permanent residences for the elderly) and day care centres	By trained professionals (neurologist and geriatrician)
		7	Charernboon T, 2019, Thailand [25]	Memory Clinic	NR
9	MMSE	6	Broche-Perez Y et al., 2018, Cuba [72]	Primary Care Community Centre: nursing homes (permanent residences for the elderly) and day care centres	By professionals (neurologist and geriatrician)
		8	Chen K-L et al., 2016, China [26]	Hospital	By trained examiner
		10	Chiu HF et al., 2017, Hong Kong [27]	Community	By untrained examiner (research assistant)
		12	Chu L et al., 2015, Hong Kong [29]	Memory Clinic, Community	By examiner
		22	Julayanont P et al., 2015, Thailand [32]	Community Hospital	By trained professionals (nurse with expertise in cognitive assessment)
		24	Phua A et al., 2017, Singapore [34]	Memory Clinic	NR
		26	Lee S et al., 2016, Australia [66]	Community, Memory Clinic	Unclear
		31	Mellor D et al., 2016, China [36]	Community	By trained professionals (psychologist or psychiatrist)
		38	Rakusa M et al., 2018, Slovenia [50]	Community	NR
		44	Van de Zande E et al., 2017, Netherlands [53]	Memory Clinic	By trained examiner
		47	Xu F et al., 2019, China [40]	Community	By trained examiner
		48	Yavuz B et al., 2017 Turkey [73]	Geriatrics Clinic	By trained examiner
		11	Chiu P et al., 2019, Taiwan [28]	Health Care Centres	By professionals (neuropsychologist)
		29	Malek-Ahmadi M et al., 2015, US [57]	Community	NR
10	CFI	9	Chipi E et al., 2017, Italy [46]	Memory Clinic	By professionals (neuropsychologist)
11	RBANS	19	Heyanka D et al., 2015 [71]	Medical Centre	NR
12	HKBC	10	Chiu HF et al., 2017, Hong Kong [27]	Community	By untrained examiner (research assistant)
13	NMD-12	11	Chiu P et al., 2019, Taiwan [28]	Health Care Centres	By professionals (neuropsychologist)
14	Qmci	13	Clarnette R et al., 2016, Australia [65]	Geriatrics Clinic	By trained professionals (geriatrician)
		48	Yavuz B et al., 2017 Turkey [73]	Geriatrics Clinic	By trained examiner
15	CCQ	14	Damin A et al., 2015 Brazil [62]	Clinical	By professionals (physician)or self-administered
16	CDT	15	Duro D et al., 2018, Portugal [47]	Tertiary Centre	By professionals (neuropsychologist)
		38	Rakusa M et al., 2018, Slovenia [50]	Community	NR
		39	Ricci M et al., 2016, Italy [51]	Memory Clinic, Community	By untrained examiner
		45	Vyhnálek M et al., 2016, Czech Republic [54]	Memory Clinic	By professionals (neuropsychologist, neurologist, resident)
17	HK-VMT	17	Fung AW-T et al., 2018, Hong Kong [30]	Community	Self-administered (touch-screen laptop)
18	TorCA	16	Freedman M et al., 2018 [70]	Suitable for use in any medical setting	By trained examineror professionals (health care professionals)
19	TICS	18	Georgakis MK et al., 2017, Greece [67]	Community, Health Centre	By professionals (health care professionals)
20	VOSP	20	Huang L et al., 2018, China [31]	Memory Clinic	By trained examiner
21	TYM	21	Iatraki E et al., 2017, Greece [68]	Rural Primary Care	By trained examiner
		44	Van de Zande E et al., 2017, Netherlands [53]	Memory Clinic, Primary Clinical Setting (GP practice, home care)	Self-administered (under supervision)
22	GPCog	21	Iatraki E et al., 2017, Greece [68]	Rural Primary Care	By trained examiner
		47	Xu F et al., 2019, China [40]	Outpatient Clinical, Primary Care	By trained examiner
23	CVLT	26	Lee S et al., 2016, Australia [66]	Community, Memory Clinic	NR
24	The Envelope Task	26	Lee S et al., 2016, Australia [66]	Community, Memory Clinic	NR
25	PRMQ	26	Lee S et al., 2016, Australia [66]	Community, Memory Clinic	NR
26	Single-item Memory Scale	26	Lee S et al., 2016, Australia [66]	Community, Memory Clinic	NR
27	FCSRT	27	Lemos R et al., 2016, Portugal [48]	Community, Hospital	NR
28	AQ	29	Malek-Ahmadi M et al., 2015, US [57]	Designed for ease of use in primary care setting	NR
29	FAQ	29	Malek-Ahmadi M et al., 2015, US [57]	Community	NR
		32	Mitchell J et al., 2015, US [59]	Community	By professionals (clinician)
30	BCAT	30	Mansbach W et al., 2016, US [58]	Long-Term Care	By professionals
31	AD8	11	Chiu P et al., 2019, Taiwan [28]	Health Care Centres	By professionals (neuropsychologist)
		30	Mansbach W et al., 2016, US [58]	Long-Term Care	Self-administered
32	DSRS	32	Mitchell J et al., 2015, US [59]	Community	By professionals (clinician)
33	CMLT	32	Mitchell J et al., 2015, US [59]	Community	By professionals (clinician)
32 + 33	CWLT-5 + DSRS	32	Mitchell J et al., 2015, US [59]	Community	By professionals (clinician)
34	BADLS	32	Mitchell J et al., 2015, US [59]	Community	By professionals (clinician)
35	DSR	33	Ni J et al., 2015, China [37]	Memory Clinic	NR
36	mSTS-MCI	34	Park J et al., 2018, South Korea [38]	Clinical settings, Primary care, Geriatrics Outpatient Clinics	By professionals (occupational therapist), using mobile application
37	CAMCog	37	Radanovic M et al., 2017, Brazil [64]	Clinical	By professionals (physician)
38	MBT	40	Roman F et al., 2016, Argentina [69]	Clinical	NR
39	SAGE	41	Scharre D et al., 2017, US [60]	Community, Clinic, Research	Self-administered (paper-based or on tablet)
40	Semantic Fleuncy/VF	42	Serna A et al., 2015, Spain [52]	Community	NR
41	Logical Memory	42	Serna A et al., 2015, Spain [52]	Community	NR
42	STMS	43	Townley R et al., 2019 US [61]	Community, Primary Care	NR
43	DMS48	46	Feng X et al., 2017, China [39]	Memory Clinic	By trained examiner
44	ADAS-Cog	49	Zainal N et al., 2016, Singapore [41]	Clinical Trials, Clinic	By trained examiner
45	IADL	11	Chiu P et al., 2019, Taiwan [28]	Health Care Centres	By professionals (neuropsychologist)
46	CASI	11	Chiu P et al., 2019, Taiwan [28]	Health Care Centres	By professionals (neuropsychologist)
47	NPI	11	Chiu P et al., 2019, Taiwan [28]	Health Care Centres	By professionals (neuropsychologist)
48	BNT	20	Huang L et al., 2018, China [31]	Memory Clinic	By trained examiner
49	STT	20	Huang L et al., 2018, China [31]	Memory Clinic	By trained examiner
50	JLO	20	Huang L et al., 2018, China [31]	Memory Clinic	By trained examiner
51	ST	20	Huang L et al., 2018, China [31]	Memory Clinic	By trained examiner
52	VCAT	23	Khandiah N et al., 2015, Singapore [33]	Community, Clinical	By trained examiner
		28	Low A et al., 2019, Singapore [35]	Community, Memory Clinic	By professionals (psychologist)

Note: ‘Article No.’ extracted from Table 2. Abbreviation list for Table 3: NR: not reported.

**Table 4:** Psychometric Properties of Cognitive Tools to Detect Mild Cognitive Decline.

No.	Cognitive Tool	Version of Tools	Author, Year, Country	Range of Total Score	Cut-Off Point *	Sn/Sp (%)	Validity		Reliability	Affecting Factors	Duration (mins)
							AUC (%)	PPV/NPV (%)			
1	6CIT	Portuguese Version	Apostolo JLA et al., 2018, Portugal [42]	8–11	≤10 (*all literacy level)*	82.78/84.84	91	84.3/83.3	High test–retest reliability, Strong internal consistency	Literacy Level	2 to 3
		Portuguese Version	Apostolo JLA et al., 2018, Portugal [42]	4–15	≤12 (*education 0–2 years)*	93.44/68.09	94	88.4/80	High test–retest reliability, Strong internal consistency	Literacy Level	2 to 3
		Portuguese Version	Apostolo JLA et al., 2018, Portugal [42]	9–12.03	≤10 (education *3–6 years)*	88/86.23	95	82.2/90.8	High test–retest reliability, Strong internal consistency	Literacy Level	2 to 3
2	EMQ	-	Avila-Villanueva M et al., 2016, Spain [43]	NR	NR	NR	NR	NR	NR	NR	NR
3	BSRT	-	Baerresen KM et al., 2015, US [55]	NR	NR	Predicted conversion to MCI and the conversion to AD				NR	NR
4	RCFT	-	Baerresen KM et al., 2015, US [55]	0–36	NR	Predicted conversion from normal aging to MCI				NR	NR
		Rey Complex Figure Test Copy (CFT-C)	Huang L et al., 2018, China [31]	0–36	≤32	46.9/76.9	81.6	NR	NR	NR	NR
5	TMT	Test B (TMT-B)	Baerresen KM et al., 2015, US [55]	NR	NR	Predicted conversion to MCI and the conversion to AD				NR	NR
6	MoCA	Czech Version (MoCA-CZ)	Bartos A et at., 2018, Czech Republic [44]	0–30	≤25	94/62	89	NR	NR	NR	12 ± 3
		Czech Version (MoCA-CZ)	Bartos A et at., 2018, Czech Republic [44]	0–30	≤24	87/72	89	NR	NR	NR	12 ± 3
		Chinese Version (MoCA-BC)	Chen K-L et al., 2016, China [26]	0–30	≤19 *(education ≤6 years)*	87.9/81	89.6	NR	NR	NR	NR
		Chinese Version (MoCA-BC)	Chen K-L et al., 2016, China [26]	0–30	≤22 *(education 7–12 years)*	92.9/91.2	94.9	NR	NR	NR	NR
		Chinese Version (MoCA-BC)	Chen K-L et al., 2016, China [26]	0–30	≤24 *(education >12 years)*	89.9/81.5	91.6	NR	NR	NR	NR
		Cantonese Version	Chiu HF et al., 2017, Hong Kong [27]	0–30	≤19/20	80/86	91.3	94/98	NR	Education	NR
6	MoCA	Cantonese Chinese Version	Chu L et al., 2015, Hong Kong [29]	0–30	22/23	78/73	95	NR	High test–retest reliability, High internal consistency, High inter-rater reliability	Education (sex and age not associated)	≤10
		-	Clarnette R et al., 2016, Australia [65]	0–30	≤23	87/80	84–92	95/58	NR	NR	NR
		Basic Version (MoCA-B)	Julayanont P et al., 2015, Thailand [32]	0–30	24/25	86/86	NR	85/82	Good internal consistency	Designed to be less dependent upon education and literacy	15 to 21
		-	Phua A et al., 2017,Singapore [34]	0–30	NR	63/77	NR	70/65	NR	NR	NR
		-	Krishnan K et al., 2016, US [56]	0–30	≤26	51/96	NR	NR	Good test–retest reliability	NR	10
6	MoCA	-	Mellor D et al., 2016, China [36]	0–30	≤22.5	87/73	89	54.5/93.6	NR	Age, Gender, Education	NR
		Brazilian Version (MoCA-BR)	Pinto T et al., 2019, Brazil [63]	0–30	NR	NR	NR	NR	Good internal consistency, Good test–retest reliability, Excellent inter-examiner reliability	NR	13.1 ± 2.7
		Italian version	Pirrotta F et al., 2014, Italy [49]	0–30	≤15.5	83/97	96	NR	High intra-rater reliability, High test–retest agreement, Excellent inter-rater reliability	NR	10
		-	Townley R et al., 2019 US [61]	0–30	≤26	89/47	Incident MCI: 70, a-MCI: 90, na- MCI: 84	NR	NR	NR	NR
6	MoCA	-	Yavuz B et al., 2017, Turkey [73]	0–30	<26	59/72	69	72/71	NR	NR	10
		-	Chiu P et al., 2019, Taiwan [28]	0–30	19/20	68/65	67	NR	NR	Age, Education	NR
		-	Huang L et al., 2018, China [31]	0–30	≤24	81.5/65.1	81.8	NR	NR	NR	NR
7	BCSE	Dutch Version	Bouman Z et al., 2015 Netherlands [45]	0–58	≤46	81/80	NR	61/92	Excellent inter-rater reliability, High internal consistency	Age	5 to 15
		Dutch Version	Bouman Z et al., 2015 Netherlands [45]	0–58	≤27	84/76	NR	57/92	Excellent inter-rater reliability, High internal consistency	Age	5 to 15
8	ACE	Cuban Revised Version (ACE-R)	Broche-Perez Y et al., 2018, Cuba [72]	0–100	≤84	89/72	93	NR	Good internal consistency reliability	Age, Years of Schooling	A few mins more than MMSE
		Thai Mini Version	Charernboon T, 2019, Thailand [25]	0–100	21/22	95/85	90	80.9/96.2	High internal consistency	NR	8 to 13
9	MMSE	-	Broche-Perez Y et al., 2018, Cuba [72]	1–30	25/26	56/83	63	NR	NR	NR	NR
		-	Chen K-L et al., 2016, China [26]	1–30	≤26	86.2/60.3	79.7	NR	NR	NR	NR
		-	Chen K-L et al., 2016, China [26]	1–30	≤27	78.6/52.2	73.6	NR	NR	NR	NR
		-	Chen K-L et al., 2016, China [26]	1–30	≤28	76.4/53.4	72.1	NR	NR	NR	NR
		Cantonese Version	Chiu HF et al., 2017, Hong Kong [27]	1–30	25/26	83/84	90.4	93/98	NR	NR	NR
9	MMSE	Chinese Version	Chu L et al., 2015, Hong Kong [29]	1–30	27/28	67/83	78	NR	NR	Education	NR
		Thai Version	Julayanont P et al., 2015, Thailand [32]	1–30	NR	33/88	70.2	NR	NR	NR	NR
		-	Phua A et al., 2017, Singapore [34]	1–30	NR	70/59	NR	64/66	NR	NR	NR
		-	Lee S et al., 2016, Australia [66]	1–30	<29	75.7/68.9	77	NR	NR	Emotional status indices (anxiety and depression)	NR
		-	Mellor D et al., 2016, China [36]	1–30	<25.5	68/83	85	60.5/87.4	NR	Age, Gender, Educational Level	NR
9	MMSE	-	Rakusa M et al., 2018, Slovenia [50]	1–30	25/26	20/93	63	NR	NR	NR	NR
		-	Van de Zande E et al., 2017, Netherlands [53]	1–30	≤23	57/98	68.5	96/69.5	NR	Education	5 to 10
		-	Xu F et al., 2019, China [40]	1–30	27 ≤ and ≤ 29	59/58.2	NR	NR	NR	NR	5 to 10
		Standardised Mini Version (SMMSE)	Yavuz B et al., 2017 Turkey [73]	1–30	≤23	36/94	71	87/56	NR	NR	NR
		-	Chiu P et al., 2019, Taiwan [28]	1–30	26/27	64/70	66	NR	NR	Age, Education	NR
		-	Malek-Ahmadi M et al., 2015, US [57]	1–30	NR	Small sensitivity to change (helpful in detecting change over time)			56% Reliability	NR	NR
10	CFI	Italian Version	Chipi E et al., 2017, Italy [46]	0–14	NR	NR	Accurate		Reliable	NR	NR
11	RBANS	-	Heyanka D et al., 2015 [71]	0–100	NR	52–93/ 35–93 (based on different subtests)	NR	16–91/ 72–94 (based on different subtests)	NR	NR	NR
12	HKBC	-	Chiu HF et al., 2017, Hong Kong [27]	0–30	21/22	90/86	95.5	94/99	Good test–retest reliability, Excellent interrater reliability, Satisfactory internal consistency	NR	7
13	NMD-12	-	Chiu P et al., 2019, Taiwan [28]	NR	1/2	87/93	94	NR	NR	NR	NR
14	Qmci	-	Clarnette R et al., 2016, Australia [65]	0–100	≤60	93/80	91–97	95/73	NR	NR	4.2
14	Qmci	Turkish Version (Qmci-TR)	Yavuz B et al., 2017 Turkey [73]	0–100	<62	67/81	80	80/68	Strong inter-rater reliability, Strong test–retest reliability	NR	3 to 5
15	CCQ	8-item CCQ (CCQ8)	Damin A et al., 2015 Brazil [62]	NR	>1	97.6/66.7	High Accuracy	78.4/95.6	NR	NR	NR
		8-item CCQ (CCQ8)	Damin A et al., 2015 Brazil [62]	NR	≥2	78/93.9	High Accuracy	94.1/77.5	NR	NR	NR
16	CDT	-	Duro D et al., 2018, Portugal [47]	0–18 (Babins System)	≤15	60/62	63.8	61/61	High inter-rater reliability	NR	NR
		-	Duro D et al., 2018, Portugal [47]	0–10 (Rouleau System)	≤9	64/58	63.5	60/62	High inter-rater reliability	NR	NR
		-	Rakusa M et al., 2018, Slovenia [50]	0–4	≤3	69/91	81	NR	NR	Age, Education	<2
16	CDT	-	Ricci M et al., 2016, Italy [51]	0–5	≤1.30	76/84	Good Diagnostic Accuracy		Excellent inter-rater reliability	NR	Very short and easy
		-	Vyhnálek M et al., 2016, Czech Republic [54]	NR	NR	62–84/47 –63	NR	NR	NR	NR	NR
17	TorCA	-	Freedman M et al., 2018 [70]	0–295	≤275	80/79	79% Accuracy		Good test–retest reliability, Adequate internal consistency	NR	Median 34
18	HK-VMT	-	Fung AW-T et al., 2018, Hong Kong [30]	0–40	21/22	86.1/75.3	79.3	NR	Good test–retest reliability	Education	15
		-	Fung AW-T et al., 2018, Hong Kong [30]	0–40	<22 *(education <6 years)*	71.1/87.3	79.3	NR	Good test–retest reliability	Education	15
18	HK-VMT	-	Fung AW-T et al., 2018, Hong Kong [30]	0–40	<25 *(education >6 years)*	71.4/76.5	79.3	NR	Good test–retest reliability	Education	15
19	TICS	-	Georgakis MK et al., 2017, Greece [67]	0–41	26/27	45.8/73.7	56.9	30.6/84.3	Adequate internal consistency, Very high test–retest reliability	Age, Education	NR
20	VOSP	Abbreviated version of the Silhouettes subtest (Silhouettes-A)	Huang L et al., 2018, China [31]	0–15	≤10	79.6/65.1	81.6	NR	High internal consistency/inter-rater reliability, Excellent test–retest reliability	Gender, Education (Unaffected by age)	3 to 5
21	TYM	Greek Version	Iatraki E et al., 2017, Greece [68]	0–50	35/36	80/77	NR	47/93	Good internal consistency	Age, Education	5 to 10
		Dutch Version	Van de Zande E et al., 2017, Netherlands [53]	0–50	≤38	74/91	79.5	87.9/79.2	Good inter-rater reliability	Education	10 to 15
22	GPCog	Greek Version of GPCog-Patient	Iatraki E et al., 2017, Greece [68]	0–9	7/8	89/61	High discrimination accuracy for high education level population; Moderate accuracy for low education level population	38/95	Good internal consistency	Age, Education	<5
		Chinese Version of 2-stage method (GPCOG-C)	Xu F et al., 2019, China [40]	GPCOG-patient: 0–9; Informant Interview: 0–9	GPCOG-patient: 5–8; Informant Interview: >4	62.3/84.6	NR	NR	NR	Unaffected by education, gender and age	4 to 6
23	CVLT	Second Edition (CVLT-II)	Lee S et al., 2016, Australia [66]	0–16	<8	82.9/93.2	94	NR	NR	Emotional status indices (anxiety and depression)	NR
24	The Envelope Task	-	Lee S et al., 2016, Australia [66]	0–4	<3	64.3/91.9	83	NR	NR	Emotional status indices (anxiety and depression)	NR
25	PRMQ	-	Lee S et al., 2016, Australia [66]	0–80	<46	50/75.7	66	NR	NR	Emotional status indices (anxiety and depression)	NR
26	Single-item Memory Scale	-	Lee S et al., 2016, Australia [66]	0–5	<3	55.7/89.2	76	NR	NR	Emotional status indices (anxiety and depression)	NR
27	FCSRT	Portuguese Version	Lemos R et al., 2016, Portugal [48]	ITR: 0–48	≤35	72/83	81.8	81/75	NR	Unaffected by literacy level	~2
		Portuguese Version	Lemos R et al., 2016, Portugal [48]	DTR: 0–16	≤12	76/80	82.4	79/77	NR	Unaffected by literacy level	~30
28	AQ	-	Malek-Ahmadi M et al., 2015, US [57]	0–27	NR	Small sensitivity to change (helpful in detecting change over time)			65% Reliability	NR	NR
29	FAQ	-	Malek-Ahmadi M et al., 2015, US [57]	0–30	NR	Small sensitivity to change (helpful in detecting change over time)			63% Reliability	NR	NR
		-	Mitchell J et al., 2015, US [59]	0–30	NR	47/82	NR	NR	NR	NR	NR
30	BCAT	Short Form (BCAT-SF)	Mansbach W et al., 2016, US [58]	0–21	≤19	82/80	86	93/57	Good internal consistency, Reliable	NR	3 to 4
31	AD8	-	Chiu P et al., 2019, Taiwan [28]	0–8	1/2	78/93	92	NR	NR	Unaffected by age, education	NR
		-	Mansbach W et al., 2016, US [58]	0–8	≥1	78/30	59	78/29	Acceptable internal consistency	NR	NR
		-	Mansbach W et al., 2016, US [58]	0–8	≥2	68/63	59	83/34	Acceptable internal consistency	NR	NR
31	AD8	-	Mansbach W et al., 2016, US [58]	0–8	≥3	47/63	59	81/27	Acceptable internal consistency	NR	NR
32	DSRS	-	Mitchell J et al., 2015, US [59]	0–51	NR	60/81	NR	NR	Good construct reliability	NR	5
33	CWLT	CERAD Word List 5-minute recall test	Mitchell J et al., 2015, US [59]	NR	NR	62/96	NR	NR	NR	NR	NR
		CWLT-3rd Trial	Mitchell J et al., 2015, US [59]	NR	NR	41/90	NR	NR	NR	NR	NR
		CWLT-Trials 1-3	Mitchell J et al., 2015, US [59]	NR	NR	57/94	NR	NR	NR	NR	NR
		CWLT-Composite	Mitchell J et al., 2015, US [59]	NR	NR	66/95	NR	NR	NR	NR	NR
32 and 33	CWLT-5 + DSRS	-	Mitchell J et al., 2015, US [59]	NR	NR	76/98	NR	NR	NR	NR	NR
34	BADLS	-	Mitchell J et al., 2015, US [59]	NR	NR	36/86	NR	NR	Good construct reliability	NR	NR
35	DSR	-	Ni J et al., 2015, China [37]	NR	≤15	100/95.9	99.8	Good diagnostic accuracy	Excellent internal consistency	NR	NR
36	mSTS-MCI	mSTS-MCI Scores	Park J et al., 2018, South Korea [38]	0–18	18/19	99/93	High Concurrent Validity		High internal consistency, High test–retest reliability	NR	15
		mSTS-MCI Reaction Time	Park J et al., 2018, South Korea [38]	0–10	13.22/13.32	100/97	High Concurrent Validity		High internal consistency, High test–retest reliability	NR	15
37	CAMCog	Briefer Version (CAMCog-Short)	Radanovic M et al., 2017, Brazil [64]	0–63	51/52 *(education >9 years)*	65.2/78.8	79.7	NR	NR	NR	NR
		Briefer Version (CAMCog-Short)	Radanovic M et al., 2017, Brazil [64]	0–63	59/60 *(education ≤8)*	70/75.5	77.3	NR	NR	NR	NR
38	MBT	Argentine Version	Roman F et al., 2016, Argentina [69]	0–32	NR	69/88	88	93/55	NR	NR	6
39	SAGE	-	Scharre D et al., 2017, US [60]	6–22	<15	71/90	88	NR	NR	NR	Median 17.5
		Digitally Translated (eSAGE)	Scharre D et al., 2017, US [60]	10–22	<16	69/86	83	NR	NR	NR	Median 16
40	Semantic Fleuncy/VF	-	Serna A et al., 2015, Spain [52]	0–17	≤10.5	53/67	72	52/75	NR	NR	1
		-	Serna A et al., 2015, Spain [52]	0–17	≤11.5	62/67	72	52/75	NR	NR	1
		-	Serna A et al., 2015, Spain [52]	0–17	≤12.5	70/56	72	48/76	NR	NR	1
41	Logical Memory	20-min Delayed Recall (DR)	Serna A et al., 2015, Spain [52]	0–6	≤2.5	43/85	71	63/72	NR	NR	20
		20-min Delayed Recall (DR)	Serna A et al., 2015, Spain [52]	0–6	≤3.5	57/71	71	54/74	NR	NR	20
41	Logical Memory	20-min Delayed Recall (DR)	Serna A et al., 2015, Spain [52]	0–6	≤4.5	78/42	71	44/77	NR	NR	20
42	STMS	-	Townley R et al., 2019 US [61]	N/A	<35	72/74	Incident MCI: 71, a-MCI: 85, na-MCI: 91	NR	NR	NR	NR
43	DMS48	-	Feng X et al., 2017, China [39]	0–48	42/43	86.6/94.2	96.6	NR	NR	Age (Unaffected by education)	Short time taking
44	ADAS-Cog	ADAS-Cog 11-item	Zainal N et al., 2016, Singapore [41]	0–70	≥4	73/69	78	90/40	Excellent internal consistency	Age	30 to 45
		ADAS-Cog 12-item	Zainal N et al., 2016, Singapore [41]	0–80	≥5	90/53	79	88/58	Excellent internal consistency	NR	30 to 45
		ADAS-Cog Episodic Memory Composite Scale	Zainal N et al., 2016, Singapore [41]	0–32	≥6	61/73	73	86/41	Excellent internal consistency	NR	30 to 45
45	IADL	-	Chiu P et al., 2019, Taiwan [28]	NR	7/8	98/27	63	NR	NR	NR	NR
46	CASI	-	Chiu P et al., 2019, Taiwan [28]	NR	82/83	68/68	72	NR	NR	Age, Education	NR
47	NPI	-	Chiu P et al., 2019, Taiwan [28]	NR	3/4	63/62	63	NR	NR	NR	NR
48	BNT	-	Huang L et al., 2018, China [31]	NR	24	70.6/55.2	67.3	NR	NR	NR	NR
49	STT	Test B (STT-B)	Huang L et al., 2018, China [31]	NR	169	50.7/80	68.3	NR	NR	NR	NR
50	JLO	-	Huang L et al., 2018, China [31]	NR	27	59.7/53.2	62	NR	NR	NR	NR
51	ST	-	Huang L et al., 2018, China [31]	NR	14	64/62.6	66.4	NR	NR	NR	NR
52	VCAT	-	Khandiah N et al., 2015, Singapore [33]	0–30	18–22	85.6/81.1	93.3	89/75.9	NR	Unaffected by language	15.7 ± 7.3
		-	Low A et al., 2019, Singapore [35]	0–30	20–24	75.4/71.1	Good construct validity	74.4/72.3	Good internal consistency	Unaffected by language and cultural background	NR

Abbreviations list for Table 4: AD: Alzheimer’s Disease; Sn/Sp: Sensitivity/Specificity; AUC: Area Under Curve; PPV/NPV: Positive Predictive Value/Negative Predictive Value.

**Table 5:** Summary of the cognitive tools performance.

Tool	Cut-Off Point	Different Versions Included	Validity	Good Reliability	Affecting Factors	Administration Time ≤15 mins	Can Be Self-Administered or Conducted by Non-Professional
**6 CIT**	≤4/10/12	✓	Good/Excellent	✓	Education	✓	✓
**EMQ**	Limited results						
**BSRT**	Limited results						
**RCFT**	≤32	✓	Fair	-	-	-	x
**TMT**	Limited results						
**MoCA**	≤26	✓	Fair/Good	✓	Education (may be affected by gender and age)	✓	✓
	≤25, ≤22.5		Good				
	≤24, ≤22, ≤19, ≤15.5		Good/Excellent				
	≤20		Fair				
**BCSE**	≤27, ≤46	✓	Fair/Good	✓	Age	✓	✓
**ACE**	≤84, ≤22	✓	Good/Excellent	✓	Age, Education	✓	x
**MMSE**	≤29, ≤27	✓	Fair	✓	Age, Education, Emotional status, Gender	✓	✓
	≤28, ≤25.5, ≤23		Fair/Good				
	≤26		Good				
**CFI**	-	✓	Good	✓	-	-	x
**RBANS**	-	-	Fair	-	-	-	-
**HKBC**	≤22	-	Excellent	✓	-	✓	✓
**NMD-12**	≤2	-	Excellent	-	-	-	x
**Qmci**	<62/≤60	✓	Good/Excellent	✓	-	✓	x
**CCQ**	>1, ≥2	✓	Good/Excellent	-	-	-	✓
**CDT**	≤15, ≤9, ≤3, ≤1.3	-	Fair/Good	✓	Age, Education	✓	✓
**TorCA**	≤275	-	Good	-	-	x	x
**HK-VMT**	<22, ≤25	-	Fair	✓	Education	✓	✓
**TICS**	≤27	-	Poor/Fair	✓	Age, Education	-	x
**VOSP**	≤10	-	Good	✓	Gender, Education	✓	x
**TYM**	≤38, ≤36	✓	Fair/Good	✓	Age, Education	✓	✓
**GPCog**	≥4, ≥8	✓	Fair/Good	✓	Inconsistent results	✓	x
**CVLT**	<8	✓	Good/Excellent	-	Emotional Status	-	-
**The Envelope Task**	<3	-	Good	-	Emotional Status	-	-
**PRMQ**	<46	-	Fair	-	Emotional Status	-	-
**Single-item Memory Scale**	<3	-	Fair/Good	-	Emotional Status	-	-
**FCSRT**	≤35, ≤12	✓	Good	-	-	x	-
**AQ**	Limited results						
**FAQ**	-	-	Poor/Good	-	-	-	-
**BCAT**	≤19	-	Good	✓	-	✓	x
**AD8**	≥1, ≥2, ≥3	-	Poor/Fair	✓	-	-	✓
**DSRS**	-	-	Fair/Good	✓	-	✓	x
**CWLT**	-	✓	Fair	-	-	-	x
**CWLT + DSRS**	-	-	Good/Excellent	-	-	-	x
**BADLS**	-	-	Poor	✓	-	-	x
**DSR**	≤15		Excellent	✓	-	-	-
**mSTS-MCI**	≤19, ≤13.32	✓	Excellent	✓	-	✓	x
**CAMCog**	≤52, ≤60	✓	Fair/Good	-	-	-	x
**MBT**	-	✓	Good	-	-	✓	-
**SAGE**	<15, <16	-	Good	-	-	x	✓
**Semantic Fluency/VF**	≤10.5, ≤11.5, ≤12.5	-	Fair	-	-	✓	-
**Logical Memory**	≤2.5, ≤3.5, ≤4.5	✓	Poor/Fair	-	-	x	-
**STMS**	<35	-	Good	-	-	-	-
**DMS48**	≤43	-	Good/Excellent	-	Age	-	x
**ADAS-Cog**	≥4, ≥5, ≥6	✓	Good/Excellent	✓	-	x	x
**IADL**	≤8	-	Poor/Fair	-	-	-	x
**CASI**	≤83	-	Fair	-	Age, Education	-	x
**NPI**	≤4	-	Fair	-	-	-	x
**BNT**	≤24	-	Fair	-	-	-	✓
**STT**	≤169	-	Fair	-	-	-	✓
**JLO**	≤27	-	Fair	-	-	-	✓
**ST**	≤14	-	Fair	-	-	-	✓
**VCAT**	18–22, 20–24	-	Good/Excellent	✓	x	x	✓

Extracted and evaluated from Table 3 and Table 4. ‘✓’ represents yes; ‘x’ represent no; ‘-’ represent unavailable data. Multiple ratings recorded if there were different results from included articles.

## Supplementary Materials

The following are available online at https://www.mdpi.com/article/10.3390/nu13113974/s1, Table S1: the final search strategy for MEDLINE.
