# Supplementary material for: Evaluation of Available Cognitive Tools Used to Measure Mild Cognitive Decline: A Scoping Review
Source: Nutrients. 2021 Nov 8;13(11):3974. doi: 10.3390/nu13113974 (PMC8623828; doi:10.3390/nu13113974)
Supplement: Supplementary file 1 [file nutrients-13-03974-s001.zip › nutrients-1442795-supplementary.pdf]

Supplementary Table S1. The final search strategy for MEDLINE.

**1**	**Dementia/or mild cognitive decline.mp. or Alzheimer Disease/or Cognitive Dysfunction/**
2	Mild cognitive impairment.mp
3	Cognitive decline.mp
4	1 or 2 or
5	Neuropsychological Tests/or battery.tw.
6	Cognit* screen* tool*.mp.
7	5 or 6
8	4 and 7
9	Limit to "middle aged (45 plus years)
10	Limit to English language
